# Subjective knee apprehension is not associated to physical parameters 6–12 months after anterior cruciate ligament reconstruction

**DOI:** 10.1186/s40634-022-00545-0

**Published:** 2022-11-07

**Authors:** Morgan Gauthier, Thanh Nam Lê, Antonia Wehn, Samuel Joseph, Philippe M. Tscholl

**Affiliations:** Department of Orthopedics and Traumatology, University Hospital of Geneva, Rue Gabrielle-Perret-Gentil 4, 1205 Geneva, Switzerland

**Keywords:** Return to sport, Test battery, Psychological readiness, Risk factors, Re-test

## Abstract

**Purpose:**

Anterior cruciate ligament (ACL) rupture is a common injury and psychological parameters measured at 6–8 months are said to be almost more predictive for return to sport (RTS) than physiological. Purpose was 1) to evaluate the correlation between knee apprehension using ACL-RSI and physical factors after ACL reconstruction (ACLR), 2) to assess the correlation between ACL-RSI and patient parameters (age, pivot-sport, BMI), and 3) to evaluate ACL-RSI over time.

**Methods:**

Patients with ACLR with or without meniscal repair between 2013 and 2020 were retrospectively analyzed. Including criteria were RTS testing battery, assessed at least 6 months after surgery, including physical parameters (strength, triple hop test, side hop test, and bilateral knee stability) and psychological parameters (ACL-RSI). 5 subgroups were analyzed to assessed factors such as age, BMI, pivot sport, time interval between two RTS testing battery.

**Results:**

Three hundred three patients (212 male, 91 female) presenting ACLR were included. Mean age at surgery was 27 (± 8) years. 258 patients practiced pivot-sport activity and 45 non-pivot-sport activity. The mean interval between ACL rupture and surgery was 6.5 (± 4.5) months. RTS testing battery were performed at 8 (± 7) months after ACLR.

Mean ACL-RSI was 58 (± 28). 1) ACL-RSI was not influenced by muscle strength, coordination and stability of the knee. 2) ACL-RSI was significantly better in lower BMI and non-pivot-sport activities. No correlation was found between graft type, age, sex, and ACL-RSI assessment. 3) For patients who performed two RTS testing battery at 8 and 12 months, ACL-RSI did not significantly increase over time (56 to 64 points, *p* = 0.22) in spite of significant increased quadriceps (127 to 151 Nm/kg, *p* = 0.005) and hamstring (93 to 105 Nm/kg, *p* = 0.05) strength.

**Conclusions:**

Psychological readiness before RTS, measured upon ACL-RSI does not correlate with any physical parameter at 8–12 months postoperatively. Although quadriceps and hamstring strength increased significantly over time, ACL-RSI does not and must therefore be routinely assessed.

## Background

Anterior cruciate ligament (ACL) rupture is a common injury in pivoting and jumping sports activities such as football, basketball or ski [1]. ACL reconstruction (ACLR) is performed with the aim to return to sport (RTS) and lower the risk of further knee injuries [1]. RTS is found in 65–82% of athletes, however only 25–50% still perform at the same level 3 years after the surgery [2, 3]. Reasons explaining not returning to sport are primarily psychological, not trusting their knee (28%), fear of a new injury (24%), and only in the third place poor knee function with insufficient subjective recovery (22%) [4]. Several measures are available to assess the psychological readiness to RTS, such as the Knee-Self Efficacy Scale, the Tampa Scale of Kinesiophobia or the Anterior Cruciate Ligament Return to Sport after Injury (ACL-RSI) scale which is a common score used in literature that assess some psychological factors as knee apprehension and fear of a new injury [5–7]. Physical parameters such as stability of the knee, inadequate rehabilitation, muscle weakness, and poor movement quality have shown to be risk factors for low RTS. It would be interesting to know if a correlation between these parameters and psychological readiness measured with ACL-RSI scale does exist, in which case, the post-operative apprehension could be influenced. Although physical factors are obviously important to assess after ACLR, we hypothesize that physical factors and psychological readiness (ACL-RSI) were not necessarily correlated.

The purposes of this study were 1) to evaluate the correlation between knee apprehension using ACL-RSI and physical factors including quadriceps and hamstring strength, coordination and anterior tibial laxity stability after ACLR, 2) to assess the correlation between ACL-RSI and socio-demographic factors such as age, pivot-sport, BMI, and 3) to evaluate ACL-RSI score over time within the first 12 months after ACLR and if physical factors do have an influence on psychological readiness.

## Methods

IRB approval for this retrospective study was received by the local ethical committee (Project-ID 2020–02201).

### Patient recruitment and surgical and rehabilitation details

Participants were recruited and data collected at the Geneva university hospitals, a primary national (sports) trauma center. In this retrospective analysis of a prospectively collected database, all patients presenting a primary ACL injury, with or without meniscal injury, treated by ACLR between 2013 and 2020 were included. Inclusion criteria were an ACL-RSI assessment at least 6 months after surgery with a concomitant physical factors assessment on the same day. Exclusion criteria included, patients lost to follow-up or without ACL-RSI assessment at least 6 months after surgery, multiligamentary lesions and concomitant bilateral ACL tear. Five senior surgeons, all fellowship trained for arthroscopic knee surgery performed ACLR using autograft only. The type of graft was used depending on surgeon preferences. Quadricipital tendon was harvesting on the same side as the injured ACL, with or without bone bloc placed in the tibial tunnel. Hamstring tendon was a 4–5 stranded semitendinosus/gracilis graft. Interference screws (MILAGRO®, De Puy Mitek, Raynham, MA, USA) were used for both femoral and tibial fixation.

The rehabilitation protocol was standardized. Patients had a preoperative rehabilitation before ACLR in order to recover the range of motion (ROM) and increase quadriceps and hamstring strength. After surgery, patients were recommended to partial weight-bearing for 2 weeks followed by progressive full weight-bearing. ROM was free, excepted if meniscus suture was performed, in such case ROM was limited in flexion–extension at 90°-0°-0° with a dynamic knee brace during 1 month.

The rest of the rehabilitation was performed step by step depending on functional criterion. First, ROM recovery, with muscular awakening. Second, stability of the pelvis and active extension during walk. Third, muscle reinforcement, unimodal balance, footing and cycling. Fourth, plyometrics. Fifth, cutting movement. Therefore, rehabilitation was not based on a specific time schedule but on patient’s progression.

Follow-up controls with the surgeon were done at 6 weeks, 3 months and 6 months.

### Assessment

Sample size calculation was performed based on an expected correlation coefficient of 0.2, an α level of 0.05 and a test power of 80%. For this, at least 194 patients were needed in our trial.

ACL-RSI assessment allowed evaluation of knee apprehension [7].

### Independent measures

#### Sport testing battery

The physical test were performed by a sport-physiotherapist and included quadriceps and hamstring strength, coordination and stability. Muscle strength, including concentric quadriceps strength (Nm/kg) at 60°/s, concentric hamstring strength (Nm/kg) at 60°/s, and eccentric hamstring strength (Nm/kg) at 90°/s, was measured with a CON-TREX® MG (Con-Trex AG, Dübendorf, Switzerland) which is an isokinetic dynamometer [8]. Moreover, Limb Symmetry Index (LSI) was calculated allowing comparison to the contralateral side. Coordination was evaluated with triple hop test and side hop test [9]. Stability of the knee was assessed by measuring anterior tibial translation using a GNRB® laximetry (Genourub, Sheng Hung Medical Co. Ltd., Taiwan), which is a robotic arthrometer designed to avoid operator-dependent errors [10]. The differential anterior–posterior laxity was calculated comparing the affected leg with the opposite leg.

#### Demographic

Patient sex, age, BMI, type of graft, time interval between the trauma and the surgery and time interval between the surgery and the ACL-RSI test were recorded from medical records.

#### Subgroup analysis

Five subgroup analysis were performed. First, a comparison was performed between patients younger than 20 years old and patients older than 20 years old in order to find a correlation between age and ACL-RSI. Second, sport activities were analyzed according to pivot-sport and non-pivot sport to define if a correlation between the type of sport and ACL-RSI does exist. Third, analysis was performed using an ACL-RSI group of less than 40 and an ACL-RSI group of more than 65 with the aim to highlight influencing factors that could explain these two extremes ACL-RSI score. Fourth, patients were divided in a group with an anterior tibial translation difference less than 1 mm and a group with more than 2 mm in order to know if a higher differential anterior–posterior laxity is a risk factor influencing ACL-RSI. Fifth, patients that performed two different RTS testing batteries at a minimal time interval of 2 months were analyzed. The aim is to evaluate ACL-RSI improvement between two RTS testing battery and if physical parameters influence ACL-RSI over time.

Results are expressed in mean and standard deviations. Analysis was performed using Pearson correlation analysis, Chi square test and paired sample two-tailed t-test. Statistical analysis were performed by using StatPlus (StatPlus version 7.6.5, Addinsoft, NY, USA). The chosen level of evidence was *p* < 0.05.

## Results

303 patients with a median age of 27 years old (± 8) were included. The mean interval between ACL rupture and surgery was 6.5 months and could be considered as late ACLR. On 245 concomitant meniscus lesions, 207 (84%) were sutured and 31 (13%) underwent partial meniscectomy. Quadricipital tendon was the most used graft (73%), with or without bone (*N* = 166 bone quadricipital tendon, *N* = 55 quadricipital tendon). The mean time interval between surgery and ACL-RSI assessment with sport testing battery was 8 (± 7) months. Mean ACL-RSI was 58 (± 28) points. Results of quadriceps and hamstring strength, coordination tests and anterior–posterior laxity are summarized in Table 1.

**Table 1:** ACL-RSI and sport testing battery after ACL reconstruction

	Patients ***n*** = 303
**Sex**	212 men (70%), 91 women (30%)
**Age (years)**	27 (± 8) years
**BMI (kg/m**^**2**^**)**	24.3 (± 4.2)
**Interval trauma – surgery (months)**	6.5 (± 4.5) months
**Meniscal tear**	245
**- Meniscal suture**	207 (84%)
**- Partial meniscectomy**	31 (13%)
**Graft type**	221 Quadricipital autograft (73%) 76 Hamstring autograft (25%) 6 Patellar autograft (2%)
**Interval surgery – test (months)**	8 (± 7)
**Knee apprehension**	
**- ACL-RSI**	58 (± 28)
**Muscle strength**	
**- Concentric quadriceps strength at 60°/s (**Nm/kg) **- Concentric hamstring strength at 60°/s (**Nm/kg) **- Eccentric hamstring strength at 90°/s (**Nm/kg)	129 (± 45) (LSI 74%) 91 (± 32) (LSI 92%) 112 (± 39) (LSI 87%)
**Coordination**	
**- Triple hop test (meters)** **- Side hop test (jumps)**	3.9 (± 1.4) (LSI: 85%) 38.7 (± 18.2) (LSI: 90%)
**Anterior tibal laxity**	
**- GNRB absolute laximetry (operative side)** **- GNRB differential laximetry to opposite side**	5.6 (± 2.6) 0.4 (± 0.2)

*BMI* Body mass index, *ACL-RSI* Anterior cruciate ligament return to sport after injury, *LSI* Limb symetry index

No correlation was observed with quadriceps strength (R = -0.09, *p* = N.S.), hamstring strength (R = -0.04, *p* = N.S.) side hop test (R = -0.03 *p* = N.S.), triple hop test (R = -0.08, *p* = N.S.) anterior–posterior differential laximetry (R = 0.03; *p* = N.S.), graft type (R = 0.04; *p* = 0.58), age (R = 0.11; *p* = N.S.), sex (R = 0.006; *p* = N.S.), time interval between ACL rupture and surgery (R = 0.04; *p* = N.S.), and time interval between surgery and ACL-RSI assessment with concomitant sport testing battery (R = 0.02; *p* = N.S.). Better ACL-RSI were observed in lower BMI (R = 0.13, *p* = 0.033) and non-pivot-sport activities (R = 0.113, *p* = 0.029).

### Younger patients (< 20 years old) versus older patients (> 20 years old)

Patients below the age of 20 (*n* = 81) had a significant higher ACL laxity on GNRB (0.8 ± 1.4 mm vs 0.3 ± 0.9 mm, p < 0.001) than patients above 20 years of age, however showed no significant difference in terms of ACL-RSI, muscle strength and jumping capacities (Table 2).

**Table 2:** Younger and older patients after ACL reconstruction

	Patients ***n*** = 303		***P*** values
	Age < 20 years old	Age > 20 years old	
Patients	***n*** = 81	***n*** = 222	
**Sex**	54 (67%) men, 27 (33%) women	158 (71%) men, 64 (29%) women	*p* = 0.54
**BMI (kg/m**^**2**^**)**	23.4 (± 4.6)	24.7 (± 3.9)	*p* = 0.02
**Pivot-sport activities**	88 (± 35) %	88 (± 36) %	*p* = 0.96
**Knee apprehension**			
**- ACL-RSI**	62 (± 29)	56 (± 29)	*p* = 0.09
**Muscle strength**			
**- Concentric quadriceps strength at 60°/s (**Nm/kg)	132 (± 44) (LSI 80%)	128 (± 46) Nm/kg (LSI 73%)	*p* = 0.42
**- Concentric hamstring strength at 60°/s (**Nm/kg)	90 (± 27) Nm/kg (LSI 97%)	92 (± 33) Nm/kg (LSI 91%)	*p* = 0.67
**- Eccentric hamstring strength at 90°/s (**Nm/kg)	108 (± 35) Nm/kg (LSI 91%)	114 (± 39) Nm/kg (LSI 87%)	*p* = 0.25
**Coordination**			
**- Triple hop test (meters)** **- Side hop test (jumps)**	3.9 (± 1.1) (90% of contralateral) 39.2 (± 17.5) (91% of contralateral)	3.9 (± 1.2) (87% of contralateral) 38.6 (± 18.6) (89% of contralateral)	*p* = 0.57 *p* = 0.80
**Anterior tibal laxity**			
**- GNRB absolute laximetry (operative side)** **- GNRB differential laximetry to opposite side**	5.8 (± 2.6) mm 0.8 (± 1.4) mm	5.5 (± 2.6) mm 0.3 (± 0.9) mm	*p* = 0.42 *p* = 0.0002

*BMI* Body mass index*, ACL-RSI* Anterior cruciate ligament return to sport after injury, *LSI* Limb symetry index

### Pivot-sport versus non-pivot sport activities

Patients performing non pivot-sport activities had a higher ACL-RSI compared to patients performing pivot-sport activities (*p* = 0.05). No significant differences were found between those two groups in term of age, BMI, muscle strength, coordination and anterior tibial translation difference on GNRB (Table 3).

**Table 3:** Pivot versus non-pivot sport activities after ACL reconstruction

	Patients ***n*** = 303		***P*** values
	Patients performing pivot-sport activity	Patients performing non-pivot-sport activitiy	
Patients	***n*** = 258	***n*** = 45	
**Age**	27 years old	29 years old	*p* = 0.21
**Sex**	193 (75%) men, 65 (25%) women	19 (42%) men, 26 (58%) women	*p* = 0.02
**BMI**	24.4 (± 4.2)	23.7 (± 3.5)	*p* = 0.36
**Knee apprehension**			
**- ACL-RSI**	58 (± 29)	68 (± 29)	*p* = 0.05
**Muscle strength**			
**- Concentric quadriceps strength at 60°/s** **- Concentric hamstring strength at 60°/s** **- Eccentric hamstring strength at 90°/s**	133 (± 49) Nm/kg (LSI 75%) 94 (± 35) Nm/kg (LSI 93%) 115 (± 41) Nm/kg (LSI 88%)	107 (± 43) Nm/kg (LSI 71%) 76 (± 30) Nm/kg (LSI 91%) 97 (± 37) Nm/kg (LSI 86%)	*p* =0.002 *p* =0.003 *p* =0.01
**Coordination**			
**- Triple hop test (meters)** **- Side hop test (jumps)**	4.1 (± 1.2) (88% of contralateral) 40.7 (± 16.8) (92% of contralateral)	3.2 (± 1.1) (86% of contralateral) 29.1 (± 17.8) (80% of contralateral)	*p* = 0.0001 *p* =0.0001
**Anterior tibal laxity**			
**- GNRB absolute laximetry (operative side)** **- GNRB differential laximetry to opposite side**	5.6 (± 2.7) mm 0.4 (± 1.1) mm	6.8 (± 2.6) mm 0.2 (± 1.0) mm	*p* = 0.56 *p* = 0.26

*BMI* Body mass index, *ACL-RSI* Anterior cruciate ligament return to sport after injury, *LSI* Limb symetry index

### Lower ACL-RSI (< 40) versus higher ACL-RSI (> 65)

Patients with ACL-RSI above 65 were younger (27 ± 8 vs 31 ± 10 yo, p < 0.002), with a lower BMI (24.1 ± 4.0 vs 25.3 ± 4.4, *p* = 0.05) compare to patients with an ACL-RSI below 40. No correlation was found with pivot-sport (*p* = N.S.), BMI (*p* = N.S.), muscle strength (*p* = N.S.), coordination (*p* = N.S.), and anterior tibial translation difference (*p *= N.S.) (Table 4).

**Table 4:** ACL-RSI < 40 versus > 65 after ACL reconstruction

	Patients ***n*** = 170 *		***P*** values
	ACL-RSI < 40	ACL-RSI > 65	
Patients	***n*** = 54	***n*** = 116	
**Age**	31 (± 10) years old	27 (± 8) years old	*p* = 0.002
**Sex**	37 (69%) men, 17 (31%) women	82 (71%) men, 34 (29%) women	*p* = 0.76
**BMI**	25.3 (± 4.4)	24.1 (± 4.0)	*p* = 0.05
**Pivot-sport activities**	95 (± 31)%	86 (± 37)%	*p* = 0.1
**Muscle strength**			
**- Concentric quadriceps strength at 60°/s** **- Concentric hamstring strength at 60°/s** **- Eccentric hamstring strength at 90°/s**	125 (± 46) Nm/kg (LSI 74%) 88 (± 29) Nm/kg (LSI 88%) 110 (± 35) Nm/kg (LSI 85%)	132 (± 41) Nm/kg (LSI 76%) 92 (± 34) Nm/kg (LSI 94%) 112 (± 41) Nm/kg (LSI 88%)	*p* = 0.22 *p* = 0.49 *p* = 0.79
**Coordination**			
**- Triple hop test (meters)** **- Side hop test (jumps)**	3.8 (± 1.1) (89% of contralateral) 32.8 (± 17.2) (98% of contralateral)	3.9 (± 1.1) (87% of contralateral) 40.2 (± 18.8) (88% of contralateral)	*p* = 0.37 *p* = 0.02
**Anterior tibal laxity**			
**- GNRB absolute laximetry (operative side)** **- GNRB differential laximetry to opposite side**	6.1 (± 2.1) mm 0.5 (± 1.2) mm	5.4 (± 3.4) mm 0.4 (± 1.0) mm	*p* = 0.2 *p* = 0.31

^*^Exclusion of 133 patients who had intermediate ACL-RSI score between 40 and 65

*BMI* Body mass index, *ACL-RSI* Anterior cruciate ligament return to sport after injury, *LSI* Limb symmetry index

### Anterior tibial translation difference less than 1 mm versus more than 2 mm

There was no significant difference between these two groups in terms of socio-demographic factors and physical parameters (Table 5).

**Table 5:** Anterior tibal laxity difference < 1 mm versus > 2 mm after ACL reconstruction

	Patients ***n*** = 175 *		
	Patients with GNRB difference < 1	Patients with GNRB difference > 2	
Patients	***n*** = 157	***n*** = 18	
**Age**	28 (± 9) years old	26 (± 9) years old	*p* = 0.25
**Sex h**	115 (73%) men, 42 (27%) women	14 (78%) men, 4 (22%) women	*p* = 0.41
**BMI**	24.0 (± 4.1)	25.1 (± 4.5)	*p* = 0.31
**Pivot-sport activities**	86 (± 36) %	95 (± 29)%	*p* = 0.29
**Knee apprehension**			
**- ACL-RSI**	59 (± 29)	57 (± 32)	*p* = 0.79
**Muscle strength**			
**- Concentric quadriceps strength at 60°/s** **- Concentric hamstring strength at 60°/s** **- Eccentric hamstring strength at 90°/s**	131 (± 40) Nm/kg (LSI 77%) 92 (± 33) Nm/kg (LSI 94%) 114 (± 41) Nm/kg (LSI 88%)	121 (± 44) Nm/kg (LSI 74%) 84 (± 31) Nm/kg (LSI 90%) 105 (± 38) Nm/kg (LSI 86%)	*p* = 0.35 *p* = 0.30 *p* = 0.37
**Coordination**			
**- Triple hop test (meters)** **- Side hop test (jumps)**	3.9 (± 1.1) (87% of contralateral) 39.5 (± 18.9) (92% of contralateral)	3.8 (± 1.2) (92% of contralateral) 37.9 (± 18.1) (87% of contralateral)	*p* = 0.70 *p* = 0.73

^*^Exclusion of 128 patients who had intermediate GNRB difference between 1 and 2 mm

*BMI* Body mass index*, ACL-RSI* Anterior cruciate ligament return to sport after injury, *LSI* Limb symetry index

### Patients with two sport-testing battery

A comparable group (57 patients) in terms of age, gender, interval between ACL rupture and ACLR had a first RTS testing battery at 8 (± 5) months and a second at 12 (± 6) months after surgery. ACL-RSI was 56 (± 25) in the 1^st^ test (58 for men, 51 for women). The overall score was increased to 64 after the 2^nd^ test (63 for men, 65 for women), however not significantly (*p* = N.S.). No significant correlation was found for sociodemographic factors and physical parameters (Table 6).

**Table 6:** Patients with 2 RTS testing battery after ACL reconstruction

	Patients ***n*** = 57 *		***P*** values
**Sex**	42 (74%) men, 15 (26%) women		
**Age**	26 years old (± 9)		
**BMI**	24.5		
**Interval trauma – surgery**	4 months		
**Graft type**	79% Quadricipital autograft 19% Hamstring autograft 2% Patellar autograft		
**Tests including ACL-RSI and sport testing battery**	**1**^**st**^ **test**	**2**^**nd**^ **test**	
**Interval surgery – test**	8 (± 5) months	12 (± 6) months	
**Knee apprehension**			
**- ACL-RSI**	56 (± 26)	64 (± 26)	*p* = 0.22
**Muscle strength**			
**- Concentric quadriceps strength at 60°/s** **- Concentric hamstring strength at 60°/s** **- Eccentric hamstring strength at 90°/s**	127 (± 45) Nm/kg (LSI 70%) 93 (± 32) Nm/kg (LSI 91%) 112 (± 39) Nm/kg (LSI 85%)	151 (± 41) Nm/kg (LSI 95%) 105 (± 31) Nm/kg (LSI 95%) 126 (± 40) Nm/kg (LSI 88%)	*p* = 0.005 *p* = 0.05 *p* = 0.11
**Coordination**			
**- Triple hop test (meters)** **- Side hop test (jumps)**	4.0 (± 1.4) (86% of contralateral) 37.7 (± 18.2) (86% of contralateral)	4.3 (± 1.3) (92% of contralateral) 42.5 (± 39.7) (93% of contralateral)	*p* = 0.29 *p* = 0.36
**Anterior tibal laxity**			
**- GNRB absolute laximetry (operative side)** **- GNRB differential laximetry to opposite side**	5.2 (± 2.6) mm 0.4 (± 1.2) mm	5.8 (± 2.8) mm 0.4 (± 0.9) mm	*p* = 0.28 *p* = 0.62

^*^Exclusion of 246 patients who only had one ACL-RSI assessment with one sport testing battery

*BMI* Body mass index*, ACL-RSI* Anterior cruciate ligament return to sport after injury, *LSI* Limb symetry index

## Discussion

Psychological readiness is a major factor in successful safe return to sport [11, 12]. Since its development in 2008, ACL-RSI score has been widely used after ACLR to assed psychological readiness [6, 13, 14]. Physical parameters analyzed in this study, such as quadriceps and hamstring strength, coordination and anterior tibial laxity were not correlated with ACL-RSI. However, current results showed a correlation between ACL-RSI, BMI and non-pivot-sport activities. Others sociodemographic factors such as age and sex did not influence ACL-RSI score. In the current study, ACL-RSI did not significantly increase over time in spite of significant increased quadriceps and hamstring strength between two RTS testing battery.

In the current study, no correlation was found between ACL-RSI score and physical parameters including muscle strength, coordination and anterior tibial translation difference after ACLR. These results correlate with the actual literature where no relationship is found between this psychological score assessing knee apprehension and physical factors [11, 15, 16]. Webster et al. observed that ACL-RSI was more strongly influence by IKDC subjective knee score than physical function (hop test limb symmetry) and that knee laxity was not correlated with psychological readiness [3].

The absence of correlation between age and ACL-RSI in this current study is consistent with previous research [4, 14]. But recent studies have shown significant correlation between age and an increased ACL-RSI [3, 11]. In a cross-sectional study including 635 patients, younger age was significantly associated with an increased ACL-RSI [6], whereas Faleide et al. found that older age was a predictor of return to preinjury level [11]. One hypothesis for this discrepancy could be the difference in patients selection with respectively on one side young athletic patients and on the other one a higher proportion of older patients performing recreational-level sports. Concerning our current study, our population sport level was not recorded and was not exclusively composed of athletes.

We found an association between ACL-RSI and BMI. The reason is unclear. Others have found association between high BMI and poorer outcome, in terms of WOMAC and SF-36 scores [17]

Patients performing non-pivot-sport showed better ACL-RSI scores at the time of their RTS testing battery. Compare to pivoting patients who had their test 255 days (± 150) after ACLR, patients without pivoting movement had their test 240 days (± 84) after ACLR (*p* = N.S). In such sports without pivoting movement, it is not surprising that knee apprehension was lessened. However, the ACL-RSI score is not sport-specific.

Patients having performed two tests showed a slight increase of ACL-RSI, not reaching statistical significance, contrary to previous studies that have found gradually increased after ACLR [13, 14]

The minimal lapse of time (2 months) between the two tests in our study might have been too short.

Since there was no significant increase of ACL-RSI with time exposure in the present study, it is not possible to conclude whether improving ACL-RSI comes with improving muscle strength or jumping ability. In our study, mean ACL-RSI score was 56 (± 26) points at 8 (± 5) months and 64 (± 26) points at 12 months (± 6) after surgery, which correlate with the current literature [3]. It has been shown that ACL-RSI is not improved by interventions after ACLR. A randomized trial showed no benefits in knee function and self-reported outcome of additional perturbation training to a rehabilitation program including strengthening, agility and secondary prevention [18]. Another study showed no improvement with advanced intensive training including plyometric exercises, dynamic knee stability exercises, lower extremity and core strengthening exercises and agility drills [19]. A systematic review found limited evidence on efficacy of psychosocial intervention for improving functional recovery [20].

ACL-RSI scale is known to be a good tool to assess patient readiness to return to sport. In a prospective study including 681 patients who underwent primary and revision isolated ACLR, ACL-RSI improved significantly over time and was significantly associated with return to sport. The optimal ACL-RSI score threshold to return to sport to the same preinjury sport at 2-years follow-up was ≥ 65 [14]

The use of ACL-RSI has some limitations, which has been established on a retrospective cohort. It assessed emotions, confidence in performance, and risk appraisal. However, other psychological aspects as motivation or depression are not evaluated. This score is not associated with re-rupture risk. Despite some limitations, ACL-RSI scale is a well-used score in the literature since its development, which allows a quick evaluation with only 12 questions on most important psychological aspects, which are emotions (5 items), confidence in performance (5 items) and risk appraisal (2 items) [7]. A shorter ACL-RSI scale has been proposed in 2018 [6]. This shorter version reduced the scale to 6 items, excluding redundant questions. The cutoff score to return to sport was 60 points for the short version, and 62 points for the full version. The cutoff score for patients who do not return to sport was 39 points for the short version and 42 points for the full version. This new ACL-RSI scale is as reliable as the initial ACL-RSI to assess psychological readiness to return to sport. Thus, this new version of the ACL-RSI might be a good alternative especially in case of busy clinical environment.

The strength of the current study is a large and heterogenic population analyzed and operated by different surgeons with different type of graft including concomitant meniscus injuries which reflect the real life in a public hospital.

The current study was subject to some limitations. As time from the surgery influences the ACL-RSI score, the time interval between ACL-RSI and physical assessments was not exactly the same for all patients, with a mean time of 8 months has to be taken in account. Because some patient presented slower progression in rehabilitation, with the inability to do jumping test, or were mentally not ready to have a full load of training, RTS testing battery were delayed. Furthermore, we did not have ACL-RSI at pre-surgery which could have be interesting to assess ACL-RSI before ACLR.

Concerning the BMI factor, we could have consider the lean mass which has an influence on knee stability [21].

Rotational knee stability was not assessed in this study, which is usually evaluated with Pivot-Shift. In this study, we only included reproductive tests using the GNRB® robotic arthrometer which only measured antero-posterior stability and not rotational stability. Even if studies have shown no correlation between anterio-posterior laxity and ACL-RSI, it is still unknown if rotational instability participate in knee apprehension.

Concomitant injuries as meniscal tears were included in the study. But we did not consider doing a subgroup, which would have been relevant, considering the higher instability and complications in post-operative in case of associated meniscal injuries.

## Conclusions

Physical parameters such as quadriceps and hamstring strength, coordination and anterior tibial laxity do not influence subjective knee apprehension at 6–12 months after ACLR. Only lower BMI and non-pivoting sport activity did correlate with higher ACL-RSI. Time does not significantly influence ACL-RSI score.

Although it is clear that physical assessment is mandatory after ACLR, psychological readiness is also to take into account during rehabilitation, especially in pivoting sports.

Psychological tests such as ACL-RSI are useful to assess the psychological status. In case of low ACL-RSI score despite good physical factors assessment, delay the RTS has to be considered in order to improve psychological readiness. Because it is multifactorial, further studies on psychological follow-up and management after ACLR are needed with the aim of improving readiness to RTS.

## Abbreviations

ACL: Anterior cruciate ligament
ACL-RSI: Anterior cruciate ligament return to sport after injury
ACLR: Anterior cruciate ligament reconstruction
BMI: Body mass index
LSI: Limb symmetry index
ROM: Range of motion
RTS: Return to sport
